# Noninvasive Instrument-based Tests for Detecting and Measuring Vitreous Inflammation in Uveitis: A Systematic Review

**DOI:** 10.1080/09273948.2020.1799038

**Published:** 2020-10-06

**Authors:** Xiaoxuan Liu, Benjamin TK Hui, Christopher Way, Sophie Beese, Ada Adriano, Pearse A Keane, David J Moore, Alastair K Denniston

**Affiliations:** a Ophthalmology Department, University Hospitals Birmingham NHS Foundation Trust, Birmingham, UK; b Academic Unit of Ophthalmology, Institute of Inflammation & Ageing, College of Medical and Dental Sciences, University of Birmingham, Birmingham UK; c Health Data Research UK, London, UK; d Musgrove Park Hospital, Taunton and Somerset NHS Foundation Trust, Taunton, UK; e Institute of Applied Health Research, College of Medical and Dental Sciences, University of Birmingham, Birmingham UK; f NIHR Biomedical Research Centre for Ophthalmology, Moorfields Eye Hospital NHS Foundation Trust and UCL Institute of Ophthalmology, UK

**Keywords:** Systematic review, uveitis, vitreous, vitreous inflammation, vitritis, diagnostic test, optical coherence tomography, retinal photography, ultrasound, imaging

## Abstract

**Purpose:**

This systematic review aims to identify instrument-based tests for quantifying vitreous inflammation in uveitis, report the test reliability and the level of correlation with clinician grading.

**Methods:**

Studies describing instrument-based tests for detecting vitreous inflammation were identified by searching bibliographic databases and trials registers. Test reliability measures and level of correlation with clinician vitreous haze grading are extracted.

**Results:**

Twelve studies describing ultrasound, optical coherence tomography (OCT), and retinal photography for detecting vitreous inflammation were included: Ultrasound was used for detection of disease features, whereas OCT and retinal photography provided quantifiable measurements. Correlation with clinician grading for OCT was 0.53–0.60 (three studies) and for retinal photography was 0.51 (1 study). Both instruments showed high inter- and intra-observer reliability (>0.70 intraclass correlation and Cohen’s kappa), where reported in four studies.

**Conclusion:**

Retinal photography and OCT are able to detect and measure vitreous inflammation. Both techniques are reliable, automatable, and warrant further evaluation.

Vitreous inflammation, or vitritis, is a clinical manifestation commonly found in posterior-segment involving uveitis. It is the hall-mark of intermediate uveitis, but is also common in panuveitis and may occur in posterior uveitis.^1,2^ Infiltration of the vitreous body with inflammatory cells and proteinaceous exudates gives a characteristic hazy appearance, reducing the clarity of structures behind it (the optic disc and retinal vessels) during fundoscopy.^3^ The clinical standard for measuring vitreous haze has been the National Eye Institute vitreous haze (NEI VH) scale since the Standardization of Uveitis Nomenclature (SUN) Workshop in 2005.^1^ Prior to the SUN workshop, three grading systems existed.^2,4,5^ The NEI VH scale is a 6-point grading system for estimating the vitreous clarity as seen through indirect ophthalmoscopy and is also referred to as the National Institute for Health (NIH) or Nussenblatt scale.^1,2^ The clinician’s estimate is compared to a standardized set of photographs and given a score of 0, +0.5, +1, +2, +3, or +4 (Table 1). This grading system has been the widely accepted standard for clinical assessment in routine care and for assessing disease outcomes in clinical trials.^6–8^ It has been adopted as part of composite measures of disease outcome for uveitis, alongside other markers of inflammation such as anterior chamber cells/flare, central macular thickness, visual function, and quality of life.^9,10^ However, there are drawbacks to clinician grading. Firstly, this method is subjective with only moderate interobserver agreement, even when assessed by experienced uveitis specialists.^11,12^ Secondly, the grading scale is non-continuous and non-linear, with large steps between each grade. Lastly, the system is poorly discriminatory for low levels of vitreous inflammation, where the need for sensitive detection of inflammatory activity to allow early clinical intervention, is greatest.^13^

**Table 1.** Standardization of uveitis nomenclature/Nussenblatt photographic grading of vitreous haze^a^

Grade	Description
0	No evidence of vitreal haze
Trace/0.5+	Slight blurring of optic disc margin
1+	Obscured view but definition to optic nerve head and retinal vessels
2+	Obscured view but definition to retinal vessels
3+	Optic nerve head visualized but borders are very blurry
4+	Obscured fundal view

^a^Nussenblatt et al. Standardization of Vitreal inflammatory Activity in Intermediate and Posterior Uveitis. Ophthalmology. 1985;92(4). Adopted with minor modifications by Jabs et al. Standardization of uveitis nomenclature for reporting clinical data. Results of the First International Workshop. Am J Ophthalmol. 2005;140(3).

More recently, measuring vitreous inflammation using instrument-based systems such as imaging devices has been proposed as a solution to some of these challenges. Several instruments, including fundus photography, ultrasound, and optical coherence tomography (OCT) have been used to visualize the vitreous body. These instrument-based methods have the theoretical advantage of being objective and automatable, and the changes detectable by each could be employed as surrogate measures of vitreous inflammation. This systematic review aims to identify all non-invasive, instrument-based tools (hereon referred to as index tests) with the ability to detect and measure vitreous inflammation in uveitis, and report the level of correlation between index tests and clinician grading, as well as the index tests’ reliability.

## Methods

This systematic review is reported according to the Preferred Reporting Items for Systematic Reviews and Meta-Analysis (PRISMA) statement.^14^ The methodology was specified in advance and the protocol registered with PROSPERO (CRD42017084168).^15^ Our search seeks to identify all index tests for detecting and quantifying vitreous inflammation. Where index tests were compared against a clinician grading system, the level of correlation was extracted. Any evaluation of test reliability, such as intra- and inter-observer reliability was also extracted.

### Search strategy

We combined free text terms and index terms reflecting the pathological finding of interest, ‘vitreous haze’ or ‘vitritis’ and the disease context ‘uveitis,’ ‘inflammation,’ ‘blood-retinal barrier,’ and ‘leak’ where possible (search strategy available in **Supplementary Materials**). Database searches were carried out in MEDLINE, Embase, Cochrane Controlled Register of Trials (CENTRAL), Center for Reviews and Dissemination Database (Health Technology Assessments and the Database of Abstracts and Reviews of Effects), Clinicaltrials.gov, WHO International Clinical Trials Registry Platform (ICTRP portal), British Library’s ZETOC, Conference Proceedings Citation Index (Web of Science), British Library Ethos, ProQuest and OpenGrey. We searched all databases from inception to December 4, 2019, with no date or language restrictions. We manually searched citations of review articles and included studies to identify additional relevant articles.

### Study selection

Two reviewers independently assessed study eligibility and resolved disagreements by consensus or by referral to a third reviewer. Studies were eligible if they described one or more index tests for detecting and measuring vitreous inflammation. Studies were not excluded based on the basis of subject age, gender, ethnicity, underlying etiology, or disease activity status. Animal studies and studies involving only healthy participants, single case reports, commentaries, and opinion articles were excluded.

### Data extraction

Two reviewers independently extracted data using a pre-specified data extraction sheet and resolved any discrepancies through consensus and referral to a third reviewer when needed. Data extracted included study design, population characteristics and disease phenotype, details of the index and reference tests, and outcomes relating to correlation between the two tests and test reliability. The full list of extracted items can be found in Table 2.

**Table 2.** Study characteristics.

Study	Study Design	Technology	Inclusion criteria	Exclusion Criteria	n Subjects	n Eyes	Age	n Male (%)	Etiology, n Eyes (%)
Oksala (1977)	Prospective	USS	AAU	nr	25	25	mean nr range 13–62	nr	nr
Haring (1988)	Prospective	UBM	Intermediate uveitis	nr	13	26	mean 32 (13–75 yrs)	4 (31)	nr
Doro (2005)	Prospective	UBM	Idiopathic typical or suspicious intermediate uveitis	nr	5	7	mean 17 (range 12–25)	2 (40)	idiopathic 7 (100)
Davis (2010)**	Retrospective	Retinal photography	Eyes with uveitis	Cataracts and prominent fundus pathology	nr	97	nr	nr	nr
Passaglia (2018)**	Retrospective	Retinal photography	Uveitis images from a clinical trial library, with varying degrees of vitreous haze and minimal/absent corneal and lens opacities	nr	nr	120	nr	nr	nr
Madow (2011)	Retrospective	Retinal photography	Patients with intermediate uveitis, posterior uveitis, or panuveitis from the MUST trial.^a^	Unreadable photographs, digital photographs, and those not yet received at the center by Feb 2009*	142	265	nr	nr	nr
Keane (2014)***	Retrospective	OCT	1) Eyes with vitreous haze secondary to intermediate, posterior, or panuveitis;2) Eyes with uveitis but with no evidence of vitreous haze; 3) Eyes without evidence of intraocular inflammation or vitreoretinal disease	nr	60	60	Uveitis with vitreous haze mean 49 (SD 18);Uveitis without vitreous haze, mean 44 (SD 14);Healthy controls, mean 67 (SD 8)	nr	Uveitis with haze: idiopathic 17 (57), BCR 5 (17), Toxoplasma 2 (7), sarcoidosis 2 (7), other 4 (12);Uveitis without haze: idiopathic 6 (50), BCR 2 (17), other 4 (33);Healthy 18 (100);
Zarranz-Ventura (2016)	Retrospective	OCT	Intermediate uveitis, posterior uveitis, or panuveitis	ERM preventing adequate transmission of light to the RPE, severe anatomic disruption of retinal integrity preventing adequate delineation of the RPE for analysis.	105	105	Uveitis eyes with vitritis mean 44 (SD 18); Uveitis eyes with no vitritis mean 47 (SD 15)	nr	Behchet’s disease 24 (23); BCR 22 (21); Sarcoidosis 14 (13); Non-differentiated 11 (10); Pars planitis 8 (8); VKH 6 (6); MS 4 (4); Other 16 (15)
Sreekantam (2017)	Retrospective	OCT	Eyes with uveitic CMO & having STTA	nr	22	22	47 (range 23–74 yrs)	5 (23)	Idiopathic 14 (63), Sarcoidosis 4 (17), TINU syndrome 1 (5), Behcet’s disease 1 (5), Reiter syndrome 1 (5), VKH disease 1 (5)
Keane (2015)***	Retrospective	OCT	1) Eyes with vitreous haze secondary to intermediate, posterior, or panuveitis;2) Eyes with uveitis but with no evidence of vitreous haze; 3) Eyes without evidence of intraocular inflammation or vitreoretinal disease	nr	60	60	Uveitis with vitreous haze mean 49 (SD 18);Uveitis without vitreous haze mean 44 (SD 14);Healthy controls mean 67 (SD 8)	nr	Uveitis with haze 30 (100): idiopathic 17 (57), BCR 5 (17), Toxoplasma 2 (7), sarcoidosis 2 (7), other 4 (12);Uveitis without haze 12 (100): idiopathic 6 (50), BCR 2 (17), other 4 (33);Healthy 18 (100);
Coric (2019)	Retrospective	OCT	Patients with uveitis and multiple sclerosis and healthy controls	**MS patients with uveitis**:- Poor scan quality- Relapse or corticosteroid treatment in month prior to baseline assessment- Pregnancy- previous neurological/psychiatric disorder- Drug or alcohol abuse- MRI abnormalities not consistent with **MSControls (as for MS patients with uveitis), plus**:- Family member with MS (first or second degree of consanguinity)- Significant MRI abnormalities	375	nr	Mean (SD): MS 52 (10) controls 49 (8)	Sex (F:M) MS 195:95 Controls 53:32	multiple sclerosis 290 (77)
Mahendradas (2017)	Prospective	OCT (using enhanced vitreous imaging technique)	Uveitis with vitreous cells	nr	33	59	Mean 43 (range 12–72)	11 (33)	Idiopathic Panuveitis 6; Intermediate uveitis 5; VKH 5TB 3; Sarcoidosis 3, Multifocal retinitis 3, Toxoplasmosis 2Posterior uveitis 2, Serpiginous choroiditis 1Idiopathic retinal vasculitis 1Sympathetic Ophthalmia 1Dengue retinitis 1

nr: not reported, MUST: Multicenter Uveitis Steroid Treatment, USS: ultrasound, UBM: ultrasound biomicroscopy, OCT: optical coherence tomography, AAU: acute anterior uveitis, BCR: birdshot chorioretinitis, TINU: tubular interstitial nephritis associated uveitis, VKH: Vogt Kayanagi Harada, MS: multiple sclerosis, ERM: epiretinal membrane, RPE: retinal pigmented epithelium, TB: tuberculosis, STTA: subtenon triamcinolone acetate, CMO: cystoid macular edema.^a^Kempen JH, Altaweel MM, Holbrook JT, Jabs DA, Sugar EA. Multicenter Uveitis Steroid Treatment Trial Research Group. The multicenter uveitis steroid treatment trial: rationale, design, and baseline characteristics. Am J Ophthalmol. 2010; 149(4):550–561. *Image data was from the MUST trial (NCT00132691), which excluded participants with inadequately controlled diabetes, Participants with uncontrolled glaucoma, advanced glaucomatous optic nerve injury, a history of scleritis; presence of an ocular toxoplasmosis scar and HIV infection or other immunodeficiency disease for which corticosteroid therapy would be contraindicated according to best medical judgment. ** Passaglia 2018 and Davis 2010 developed grading systems using the same set of fundus images. *** Keane 2014 and Keane 2015 used the same image dataset for both studies.

**Table 2.** (Continued).

Study	Technology	Manufacturer model	Acquisition/Image processing protocol	Area/volume	Analysis software	Automation
Index test characteristics						
Oksala (1977)	USS	Model 7100 a (Kretztechnik, Austria)	*“The transducer was pressed against the sclera and the beam aimed at the vitreous from several different directions”*	Whole axial length	na	Manual
Haring (1988)	UBM	UBM 840, (Zeiss-Humphrey, San Leandro, CA, USA)	UBM using 50 MHz probe. Gain was 80 dB: approximate spatial resolution 50 µm and penetration depth 5 mm. *“During each examination radial scanning of the anterior chamber angle/ciliary body region and the pars plana and peripheral retina was conducted in all clock hours, but with emphasis on the lower circumference of the eye.”*	nr	na	Manual
Doro (2005)	UBM	Model P45 (Paradigm Medical Industries, Salt Lake City, Utah, USA): 50-MHz probe and Cinescan S (Quantel Medical, Clermont-Ferrand, France): 20-MHz immersion open probe.	nr	nr	na	Manual
Davis (2010)**	Retinal photography	30° Zeiss fundus camera model FF4 (Carl Zeiss Meditec, Inc, Pleasanton, California, USA) with a Nikon film camera (Nikon Instruments Inc, Melville, New York, USA)	Photos modified by the application of a Bangerter occlusion filter. Films were then digitized with Nikon film scanner at 24-bit color and resolution of 300 dpi and images saved as TIF format	30° photograph	na	Manual
Passaglia (2018)**	Retinal photography	nr	Images stored as TIF format cropped to an area of 512 by 512 pixels centered on the macula	na	Custom software	Fully automated
Madow (2011)	Retinal photography	nr	Images digitized with Nikon Coolscan film scanner at 300 dpi and saved as TIF format	nr	na	Manual
Keane (2014)***	OCT	Heidelberg SPECTRALIS OCT	3–5 B-scans passing through the foveal central subfield	nr	OCTOR custom software	Semi-automatic
Zarranz-Ventura (2016)	OCT	Cirrus HD-OCT (Carl Zeiss Meditec, Dublin, California, USA)	“Macular Cube” protocol: 128 horizontal B-scans. This covers a 6 by 6 mm area.	36 mm^2^	OCTOR custom software	Semi-automatic
Sreekantam (2017)	OCT	Heidelberg SPECTRALIS OCT	Volume scan images of 20° by 20°,containing a minimum of 25 OCT B-scans on TruTrack Active and Auto Rescan follow up modes active	20° by 20°	OCTOR custom software	Semi-automatic
Keane (2015)***	OCT	Heidelberg SPECTRALIS OCT	Volume scans centered on the fovea.	nr	VITAN custom software	Fully automated
Coric (2019)	OCT	Heidelberg SPECTRALIS OCT	Macular volume scan centered around the fovea (20x20°, 512 A-scans, 49 B-scans, vertical alignment, automatic real time 16)	20° by 20°	VITAN custom software	Fully automated
Madendradas (2017)	OCT (using enhanced vitreous imaging technique)	Heidelberg SPECTRALIS OCT	Four sets of horizontal and vertical 9 mm B scans with ART 100. Enhanced Depth Imaging: position of scan shifted to lower half of screen Combined Depth Imaging: position of scan shifted to middle of screen Enhanced Vitreous Imaging: +2 diopters added to the focus	na	na	Manual

nr: not reported, USS: ultrasound, UBM: ultrasound biomicroscope, OCT: optical coherence tomography, ART: automated real-time averaging.** Passaglia 2018 and Davis 2010 developed grading systems using the same set of fundus images.*** Keane 2014 and Keane 2015 used the same image dataset for both studies.

**Table 2.** (Continued).

Study	Technology	Automation	Comparator	Eyes per grade (n)	Correlation (LCI, UCI)	Reliability test	Reliability test result
Index test correlation with clinical grading and reliability							
Oksala (1977)	USS	Manual	none	na	na	nr	na
Haring (1988)	UBM	Manual	Slit lamp fundoscopy	na	na	nr	na
Doro (2005)	UBM	Manual	none	na	na	nr	na
Davis (2010)**	Retinal photography	Manual	none	na	na	ICC	0.88 (Interobserver)
Passaglia (2018)**	Retinal photography	Fully automatic	NIH scale (6 point photographic scale), Miami scale (9 level photographic scale)	nr	Exact agreement: Cohen’s K = 0.61 (NIH scale) and 0.67 (Miami scale), Within-one level agreement: K = 0.78 (NIH scale) and 0.82 Miami scale) Within-two level agreement: K = 0.80 (NIH scale) and 0.84 (Miami scale).	nr	na
Madow (2011)	Retinal photography	Manual	NEI VH scale	0 (85) 1+ (135) 2+ (35) 3+ (9) 4+(1)	(correlation test nr) r = 0.51	ICC	0.87 (Interobserver); 0.84–0.93 (Intraobserver);
Keane (2014)***	OCT	Semi-automatic	NEI VH Grade	0, 12 0.5+, 4 1+, 13 2+, 10 3+, 3 4+, 0	Spearman’s r = 0.57	BA 95% limits of agreement between two graders. Where,“uveitis with vitreous haze” median measurement = 0.150 (IQR = 0.135) “uveitis with no VH/normal” Median measurement = 0.0767 (IQR = 0.048)	All: 0.035 Uveitis with vitreous haze (grade 0.5+ and above) 0.045; Healthy control (grade 0): 0.023;
Zarranz-Ventura (2016)	OCT	Semi-automatic	NEI VH Grade	0, 54 0.5+, 21 1+, 18 2+, 9 3+, 3 4+, 0	Spearman’s r = 0.53	nr	na
Sreekantam (2017)	OCT	Semi-automatic	none	na	na	nr	na
Keane (2015)***	OCT	fully automated	NEI VH Grade	0, 12 0.5+, 4 1+, 13 2+, 10 3+, 3 4+, 0	Spearman’s r: Vitreous:RPE signal ratio = 0.59Vitreous:RPE textural ratio = 0.60	nr	na
Coric (2019)	OCT	Fully automatic	none	na	na	nr	na
Mahendradas (2017)	OCT (using enhanced vitreous imaging technique)	Manual	Each image graded for posterior vitreous visualization: 0 = not visible 1 = barely visible 2 = clearly visible	na	na	interobserver agreement (Cohen k) of visualization scoring	Conventional OCT: 0.771 EDI 0.732 CDI 0.722 EVI 0.743

na: not applicable, nr: not reported, USS: ultrasound, UBM: ultrasound biomicroscope, OCT: optical coherence tomography, NIH: national institute of health, NEI: national eye institute, ICC: intraclass correlation, BA: bland-altman, VH: vitreous haze, IQR: interquartile range, EDI: enhanced depth imaging, CDI: combined depth imaging, EVI: enhanced vitreous imaging ** Passaglia 2018 and Davis 2010 developed grading systems using the same set of fundus images. *** Keane 2014 and Keane 2015 used the same image dataset for both studies. LCI (lower confidence interval) and UCI (upper confidence interval) was not reported in all studies.

#### Risk of Bias Assessment

Relevant features of the Quality Assessment of Diagnostic Accuracy Studies tool (QUADAS-2) were used to assess for bias in the studies. The assessment considered patient selection (if the patients receiving the index and reference tests were representative of uveitis patients and the spectrum of uveitic subtypes), index test (if the index test was interpreted without knowledge of the reference test), reference test (if the reference test was interpreted without knowledge of the index test) and flow and timing (if all patients received both tests within an appropriate time interval – within same day assessment was deemed sufficient to ensure the inflammatory status of the eye had not changed). Not all elements of QUADAS-2 were applicable. For example, “whether the reference standard is likely to correctly quantify the target disease (vitreous inflammation)” would be marked unclear for all studies, due to the known poor reliability of clinician grading. As QUADAS-2 is only applicable for studies comparing an index test to reference test, the assessment was only carried out in studies evaluating correlation between the two tests and not in studies evaluating index test reliability.

### Data analysis

For each index test, we tabulated the extracted information and provided a narrative synthesis of methodological characteristics and index tests evaluated. Studies which compared index test measurements with a reference test (such as clinician grading) and reported a correlation coefficient were included in the analysis. In these studies, where confidence intervals for correlation coefficients were not reported, correlation coefficients were normalized using Fisher’s Z transformation for meta-analysis and back transformed and presented on a forest plot for visualization only. All statistical analyses were performed using Stata Statistical Software (Release 15. College Station, TX: StataCorp LP). Meta-analysis was not performed for test correlation or reliability due to heterogeneity between studies.

## Results

### Results of the Search

**Figure 1.**
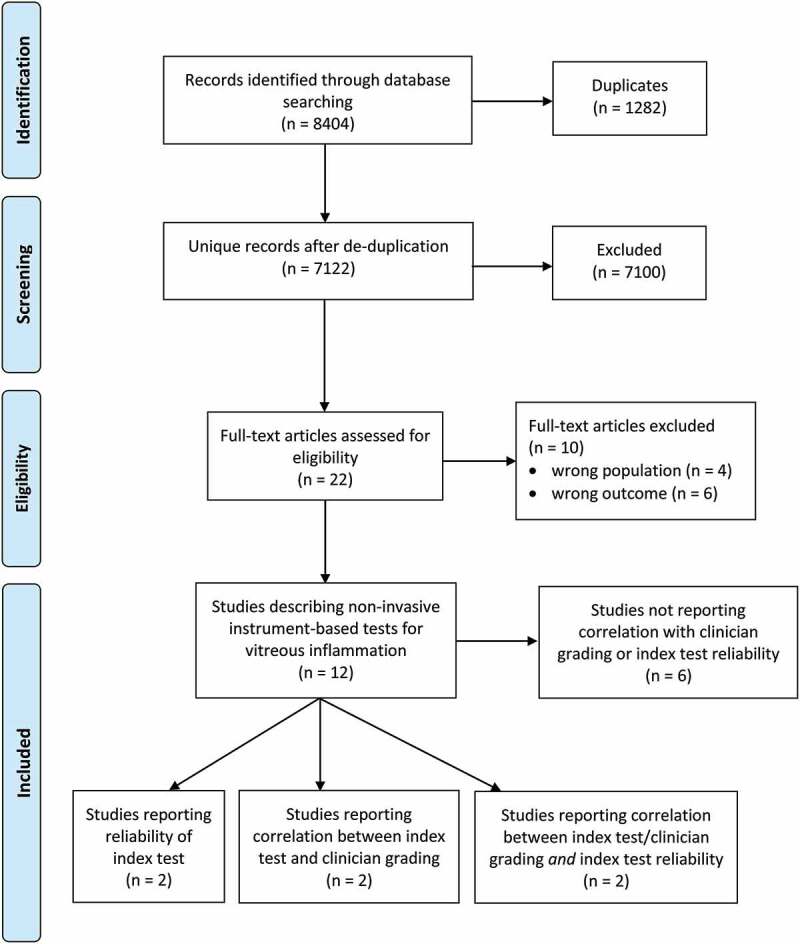
PRISMA flow diagram.

The study selection process is summarized in the PRISMA flow diagram (Figure 1).

The search yielded 7122 unique bibliographic records after removal of duplicates. Of these, 7100 were excluded based on screening of titles and abstracts. The large number of excluded records was due to the unrestrictive nature of the search strategy, which was deliberately wide, to ensure full capture of all potentially relevant technologies. The remaining 22 articles were reviewed in full text and further 10 articles were excluded. The reasons for exclusion were due to not matching the criteria for outcome (n = 6) or target population (n = 4). Twelve articles were included; two studies compared an index test with a clinician grading system, two reported test reliability, and two did both. Six studies described index tests but did not report correlation with clinician grading or index test reliability (Table 2).

### Participants’ characteristics and study design

The 12 studies included a total of at least 840 participants^16-25^ (two studies did not report the number of participants^26,27^) and at least 846 eyes (one study did not report the number of eyes.^23^) The studies were published between 1977 and 2019. Four studies were conducted prospectively^16,17,24,25^ and eight were retrospective.^18-,23-,26-,27^ Only five studies reported gender, with 29% of participants (n = 149) being male.^16,17,22-24^ The age of participants in studies ranged from 12 to 75 years.^16,17,19-25^ Five studies included mixed etiologies, including toxoplasmosis, sarcoidosis, Behcet’s disease, Birdshot chorioretinopathy, pars planitis, Vogt-Koyanagi-Harada disease, multiple sclerosis, tubulointerstitial nephritis and uveitis syndrome, Reiter’s syndrome, multifocal retinitis, serpiginous choroiditis, idiopathic retinal vasculitis, sympathetic ophthalmia, and Dengue retinitis.^19–22,24^ Two studies were narrower in their inclusion criteria with one study including intermediate uveitis only^17^ and the other including patients with multiple sclerosis only^23^. Five studies did not specify the underlying etiology of participants.^16,25-27^

### Clinical reference test

Six out of 12 studies did not compare an index test against a comparator.^17,22-26^ One study compared ultrasound biomicroscopy against qualitative features on slit-lamp fundoscopy.^16^ Four studies compared OCT^19-21^ and retinal photography^27^ against the NEI VH scale only, and one study compared retinal photography against both NEI VH scale and a photographic scale called the Miami scale (described in next section).^27^

### Instruments for detecting and quantifying vitreous inflammation

Three types of technologies with the ability to detect and quantify vitreous inflammation were identified from the 12 studies: ultrasound, retinal photography, and OCT.

Three studies employed ultrasound. One study used an A-scan instrument, model 7100A (Kretztechnik, Austria) with a transducer of 6 MHz/S mm^25^ and two studies used ultrasound biomicroscopy (the UBM 840 (Zeiss-Humphrey, San Leandro, CA, USA) with a 50 MHz probe in one study^16^ and the Model P45 (Paradigm Medical Industries, Salt Lake City, Utah, USA) with a 50 MHz probe plus the Cinescan S (Quantel Medical, Clermonth-Ferrand, France) with a 20 MHz immersion open probe in another study.^17^) The images in all three studies were interpreted manually and qualitatively, by the operator, in real-time.

Three studies used retinal photography. Davis et al. developed a 9-point scale using calibrated Bangerter filters to blur fundus photographs, originally acquired using 30⁰ Zeiss fundus camera model FF4 (Carl Zeiss Meditec Inc, Pleasanton, California, USA) with a Nikon film camera (Nikon Instruments Inc, Melville, New York, USA).^26^ This 9-point scale is known as the Miami scale and is designed to be a reference for manual clinician grading of fundus photographs. The authors tested the use of this reference scale using film fundus photographs from an imaging archive (unspecified camera and system). Madow et al. used fundus photographs originally acquired as color film slides for the MUST trial^12^ and digitized them using Nikon Coolscan film scanner (Nikon, Inc, Melville, New York, USA) at 300 dpi and saved as TIFF format.^18^ Madow et al. used the Miami scale developed by Davis et al. to grade the severity of vitreous haze in these photographs.^18^ Passaglia et al. applied an automated retinal photography analysis software to grade fundus photographs from a clinical trial library (unspecified source, camera, and system) according to the NEI VH and Miami VH scales.^27^

Six studies used OCT. Five studies used the Heidelberg SPECTRALIS OCT^19,20,22-24^ and one used the Cirrus HD-OCT (Carl Zeiss Meditec, Dublin, California, USA).^21^ Two studies used the same semi-automated image analysis technique (custom OCTOR software),^19,22^ two used the same fully automated image analysis technique (custom VITAN, which employs the same principles of pixel intensity as OCTOR, requires no manual input other than confirmation of the selected vitreous area)^20,23^ and one study used manual analysis of OCT images using a subjective observer-based grading system consisting of grades 0–2, where grade 0 was ‘not visible,’ grade 1 was ‘barely visible,’ and grade 2 was ‘clearly visible.^24^

### Index test reliability

Four studies reported index test reliability using varying methodologies. Davis et al. reported an intraclass correlation (ICC) of 0.88 between two observers grading fundus photographs against the 9-point Miami scale.^26^ Madow et al. reported an inter-observer ICC of 0.87 and an intra-observer ICC of between 0.84 and 0.93 against the Miami scale.^18^ Keane et al. used Bland–Altman plots to assess interobserver variability and reported a median 95% limits of agreement (LoA) of 0.0353 for all OCTs, 0.0450 in OCTs of uveitic eyes with vitreous haze and 0.0226 for OCTs of healthy eyes or uveitic eyes without vitreous haze. They reported the variance ratio (*F* statistic) as non-significant between groups, suggesting the measurement variance was similar in eyes with and without vitreous inflammation.^19^ Mahendradas et al. reported interobserver agreement as Cohen’s kappa >0.7 for all four tested techniques (standard OCT, enhanced vitreous imaging, enhanced depth imaging, and combined depth imaging).^24^

### Correlation between index tests and the clinical reference test: Slit-lamp based clinician grading

**Figure 2.**
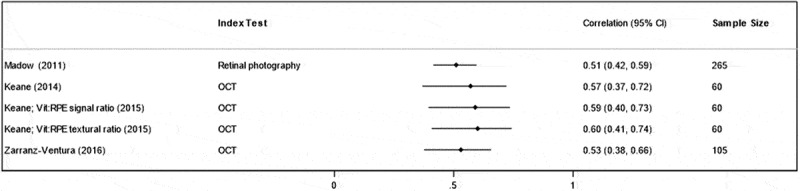
Level of correlation between index tests and clinician grading (SUN/NEI/Nussenblatt vitreous haze scale).

Four studies reported correlation between an index test and clinician grading of vitreous inflammation (three studies using OCT^19-21^ and one study using retinal photography.^18^) All studies reporting correlation used the NEI VH scale as a comparator. The total number of participants included in these four studies was 307 (430 eyes). Spearman’s *r* was used by all studies except by Madow et al. to measure the association between index test measurements and the NEI VH scale. The level of correlation between OCT measurements and the NEI VH scale using the semi-automated OCTOR software was 0.53–0.57,^19,21^ whereas for the fully automated VITAN software correlation was marginally higher at 0.59–0.60.^20,28^ Both studies by Keane et al., reporting the use of OCTOR and VITAN, used the same retrospective dataset of images. The level of correlation between manual grading of retinal photographs (using the Miami scale) versus clinician examination (using the NEI VH scale) was reported as *r* = 0.51. The correlation between index tests and the NEI VH scale are shown in Figure 2. None of the four studies reported confidence intervals for correlation coefficients and those shown in the forest plot were estimated using sample size and correlation coefficient. Passaglia et al. measured agreement between automated fundus photography grading (using the Miami scale) and clinician grading, rather than correlation. They report exact agreement, agreement within one level and agreement within two levels of 0.61, 0.78, and 0.80, respectively, against clinician grading using the NIH scale and 0.67, 0.82, and 0.84, respectively, against the clinician grading using the Miami scale.^27^

RPE: retinal pigmented epithelium, OCT: optical coherence tomography. *Keane 2015 uses VITAN, an automated version of the previously published OCTOR software. Two variations of the same technique are presented: vitreous:RPE signal ratio and vitreous:RPE textural ratio.** Keane 2014 and Keane 2015 used the same cohort of patients for both studies.

#### Risk of Bias Assessment

The patient cohorts in the correlation studies were a mixture of uveitis etiologies with a low risk of spectrum bias, except in the retinal photography study by Madow et al., where the risk was not assessable as the underlying etiology was not reported.18 Other than Madow et al., all studies used automated/semi-automated systems to quantify vitreous haze; therefore, it was assumed there was no potential influence from knowledge of the clinician grading. All studies used previously recorded clinician grading (from clinical care or clinical trials data), therefore there was no possibility that the reference test could have been influenced by the index test, which was conducted afterward. Madow et al. did not report whether the fundus photograph readers were blinded to the clinician grading results. Although the time interval between index and reference tests were not explicitly reported by any of the studies, it is presumed that clinician grading and the images acquired were performed on the same visit in all studies, even if image analysis for index tests were done at a later date.

### Study heterogeneity

After accounting for overlap between studies in terms of similar imaging techniques and duplicated patient cohorts, there was considerable heterogeneity between the methodology and populations across the included studies. Given this level of heterogeneity, we have not performed any meta-analysis of correlation or test reliability for index tests.

## Discussion

This is the first systematic review for identifying noninvasive instrument-based tests for detecting and measuring vitreous inflammation in uveitis. Three technologies were found: ultrasound, retinal photography, and OCT. Ultrasound remains primarily for qualitative assessment of features in the vitreous body and has not been shown to quantify inflammation. Retinal photography and OCT have demonstrated the most potential as methods for quantifying vitreous inflammation through automated and semi-automated means of image analysis. However, only 12 studies have been undertaken and even fewer provided sufficient evidence on test reliability or correlation with clinician grading.

Davis et al. and Madow et al. reported good interobserver reliability (ICC>0.84) and moderate correlation (*r* = 0.51) of manual grading using retinal photography (assessed using the Miami scale).^18,26^ This photographic method introduces two advantages beyond the traditional indirect biomicroscopic approach (assessed against the NEI VH scale). Firstly, it captures an adequate view of the fundus and removes the variability introduced by the level of the indirect biomicroscopy skills of the examiner. Secondly, it is based on a 9-point scale rather than the 6-point NEI VH scale, allowing smaller differences to be captured between grades. The automated retinal photography technique applied by Passaglia et al. brings added objectivity beyond the direct biomicroscopic assessment of the NEI VH scale or the original subjective photograph-to-photograph comparison of the Miami grading. On the other hand, the OCT-based technique utilizes signal intensity detected in the vitreous, to derive a measure of light reflectivity as a continuous variable. The ability to detect vitreous reflectivity on a continuous scale means the OCT-based method may potentially offer sensitivity to even smaller, but potentially clinically significant, changes in vitreous inflammation.

Whilst automation of image analysis may improve reliability, we did not find that it consistently improves correlation with clinician grading. The fully automated VITAN OCT algorithm was tested on the same dataset as the semi-automated OCTOR algorithm and showed marginally higher correlation when compared to the NEI VH grade (*r* = 0.60 versus 0.57).^20^ Manual grading of retinal photography showed moderate correlation (*r = *0.51)^18^ when compared to the NEI VH scale, similar to the moderate agreement reported for fully automated photographic grading (Cohen’s K = 0.61).^27^

## Strengths and limitations of the review

This review represents the first systematic evaluation of technologies for measuring vitreous inflammation in uveitis. The search strategy was designed to be highly sensitive, using a broad range of databases, including conference proceedings, dissertation databases and the grey literature. This review also has several limitations. An issue in undertaking systematic reviews of correlation between assessment methods is the absence of an adequate specific tool for assessing risk of bias in studies. We have used relevant elements of the QUADAS-2 tool for risk of bias assessment in test accuracy studies for the correlation studies only, where one test was being compared against another. However, this method of assessing risk of bias could not be applied to include studies which only evaluated one test (i.e. for index test reliability). Second, although we included all studies reporting instruments with the potential to detect and measure vitreous inflammation, the data extraction and analysis were focused on test reliability or correlation with the clinical standard. As a result, two studies that provide evidence of the clinical validity and value of new techniques were not discussed in detail.^22,23^ These include Sreekantam et al.’s study which reported a highly statistically significant reduction of OCT-based vitreous signal (using OCTOR) pre- and post-injection of sub-tenon’s triamcinolone in patients with uveitic macular edema, demonstrating the potential clinical utility of this technique for detecting treatment response and its potentially superior sensitivity for measuring change over the clinician based grading system; however, this study did not include NEI VH scale as a comparator.^22^ Coric et al. also explored whether a difference in vitreous intensity could be detected in patients with multiple sclerosis versus healthy controls, but did not find a measurable difference; again this study did not include NEI VH scale as a comparator.^23^ Additional imaging techniques such as ultra-wide field fundus photography (using the Optos ultra-wide field camera) have also been used to detect presence and absence of vitreous haze through manual observation.^29^ Third, the focus of this review was on correlation with the reference test. Whilst correlation is helpful in early validation, it is limited to demonstrating agreement and non-inferiority to the comparator. From correlation, it is not possible to determine if the index test is more accurate than the reference test. To determine accuracy, a more reliable reference test than the NEI VH scale is required, such as the use of invasive vitreous sampling to determine the level of protein and cellular infiltrates in the vitreous. Due to risks involved, it is unlikely that vitreous sampling will be ethically justifiable in routine practice. In the absence of a reliable reference test, future work could compare the ability of index tests versus clinician grading to detect changes in inflammation, such as the pre- and post-treatment comparison Sreekantam et al. conducted.^22^ The ability to demonstrate higher sensitivity to small changes as well as stronger association with other inflammatory markers (such as central macular thickness) and visual function, would provide further evidence of accuracy in measuring the true disease state.

## Limitations of the evidence

Firstly, due to the small number of included studies and heterogeneity in study design, meta-analyses of correlation or reliability were not possible. Several studies were conducted by the same author groups and presented sequential updates of the same technique using different approaches to image analysis, including automation.^20,27^ Most studies used retrospectively collected images, with several applying newer analysis techniques to the same image set. Incomplete reporting and varying methodology of the included studies also meant we were unable to pool estimates of correlation between index and reference tests. Secondly, authors sometimes reported correlation coefficients estimated from a mixed cohort of uveitic and healthy eyes. With the exception of Madow et al., where only uveitic eyes were included in the study, all other studies reporting correlation coefficients were a mixture of healthy and uveitic eyes.^19–21^ It was not possible to separate the two cohorts as correlation was reported at an aggregated level in all cases. On the other hand, all studies reporting intra/inter-observer reliability included uveitic eyes only. Thirdly, of those studies that reported NEI VH grading, no patients for OCT and only one patient for retinal photography had grade 4+.^18^ It could be that in dense vitreous haze, neither OCT nor photography can successfully acquire a usable image and such cases could have been excluded on the basis of poor image quality. However, it is unclear how those index tests performed in the most severe grades of vitreous inflammation.

## Clinical relevance and impact

Of the instrument-based tests identified, OCT and retinal photography are presented with the most supporting evidence in this review. Both instruments offer the attractiveness of being technologies already widely available in ophthalmic clinics. Additionally, both techniques can be combined with automated image analysis techniques. OCT additionally offers a measurement which can be continuous and it has also been shown to be sensitive to respond to treatment.^22^ At this stage there are only a few reports identified for either technology and these reports were mostly retrospective studies with small numbers of subjects. As noted earlier very few patients with severe vitritis are included in these studies, and it is difficult to draw conclusions on the validity of both instruments in the most severe levels of inflammation. It could be argued that, where inflammation is obviously detectable through clinical examination, there is less additional value of quantification by a noninvasive imaging technique. However, clearly, the ideal scenario is to have a technique that is sensitive to changes at both ends of the scale, including detecting worsening or improvement in severe inflammation.

Another major consideration is around generalizability of the study findings in the presence of ocular co-pathology. Of particular concern is media opacity such as cataract, which may cause a similar hazy appearance on fundoscopy and which could degrade image quality on both retinal photography and OCT. Given cataracts are a major complication of chronic intraocular inflammation and ocular steroid therapy, many patients with posterior uveitis have cataracts.^30^ In the included studies of this review, only Davis et al. reported the exclusion of subjects with cataracts.^26^ Zarranz-Ventura et al. assessed the use of OCT of patients with uveitis, which also included pseudophakia and patients who had undergone vitrectomy. They demonstrated no observable difference in the measurement for each of these groups compared to phakic and non-vitrectomised eyes, respectively.^21^

An important area for future work is to evaluate the relationship between instrument-based measures and visual function. Sreekantam et al. reported a correlation coefficient of 0.70 between VIT/RPE-relative intensity and visual acuity, a stronger correlation than was demonstrated when the same OCTOR technique was compared to the NEI VH grading by *Keane* et al. (*r* = 0.60).^19,22^ However, this is not a direct comparison due to different subjects in each study. No other studies explored the association between the index test measurements and visual acuity or any other measure of visual function. Whilst the relationship of visual function to inflammatory activity is complex, often being delayed and indirect, it is worthy of exploration. These tests will be of greatest value if their use enables better control of inflammation, such that vision is maintained in the immediate and long term. It is worth noting that the importance of demonstrating clinical validity through association with visual function was emphasized by regulatory bodies at the American Uveitis Society workshop at the University of California Los Angeles (UCLA) in March 2019 on *Objective Measures of Intraocular Inflammation for Use in Clinical Trials*.^31^

If the unreliability of the current reference standard is limiting the evaluation and adoption of emerging techniques, are there any other techniques we should consider as a reference test? As previously discussed, direct sampling of vitreous is unlikely to be ethically justifiable unless it is being done for diagnostic purposes. More invasive tests to quantify vitreous inflammation also exist but are largely unused. Vitreous fluorophotometry is an intravenous fluorescein-based imaging technique which can quantify leakage of dye from the blood-retinal-barrier (BRB) and has been used in the assessment of inflammation of the posterior segment.^32^ Vitreous fluorophotometry measures leakage by measuring the degree of fluorescence throughout the eye at defined axial points before and after the intravenous injection of fluorescein. It relies on the principle that the amount of leakage is proportional to the degree of BRB breakdown. However, due to its invasive nature, vitreous fluorophotometry is rarely performed and for the most part, has been used as an experimental technique rather than for clinical care.^33^ Nonetheless, it is worth considering that invasive tests like fluorophotometry may be more direct measures of inflammatory activity and may serve as better reference tests with which to validate newer noninvasive tests. Assuming invasive approaches are not undertaken, evidence supporting new techniques and eventual adoption as a ‘reference standard’ is likely to depend on demonstrating high test reliability, strong association with other evidence of inflammation (such as macular thickness, presence of vitreous cells and other vitreous inflammatory infiltrates, presence of retinal vasculitis and vascular leakage and new active inflammatory lesions), and association with visual function (recognizing that this may not be direct or immediate).

## Conclusion

Non-invasive instrument-based tests for measuring vitreous inflammation have the potential to improve reliability and speed compared to clinician grading using indirect ophthalmoscopy. Retinal photography and OCT are two promising technologies with the potential to quantify vitreous inflammation; however, further evidence beyond the proof-of-concept studies identified by this review are required to demonstrate clinical utility. Further evaluation in prospective studies should explore association with other measures of posterior-segment inflammation as well as visual function.

## Supplementary Material

Supplemental Material
