# Supplementary material for: Noninvasive Instrument-based Tests for Detecting and Measuring Vitreous Inflammation in Uveitis: A Systematic Review
Source: Ocul Immunol Inflamm. 2020 Oct 6;30(1):137–48. doi: 10.1080/09273948.2020.1799038 (PMC8935946; doi:10.1080/09273948.2020.1799038)
Supplement: Supplemental Material [file IOII_A_1799038_SM6237.docx]

# Appendix 1.

# MEDLINE Sample Search Strategy

| 1 | Exp Uveitis/ |
| --- | --- |
| 2 | Uveiti*.ti,ab. |
| 3 | Inflamm*.ti,ab. |
| 4 | Leak*.ti,ab |
| 5 | Blood retinal barrier.ti,ab. |
| 6 | 1 or 2 or 3 or 4 or 5 |
| 7 | vitreous.ti,ab. |
| 8 | Vitreous haze.ti,ab. |
| 9 | vitritis.ti,ab. |
| 10 | 7 or 8 or 9 |
| 11 | 6 and 10 |
